# A social consensus to prioritize humanization strategies for Mental Health in Castilla y León

**DOI:** 10.1192/j.eurpsy.2023.1925

**Published:** 2023-07-19

**Authors:** J. M. Pelayo-Terán, Y. Zapico-Merayo, S. Vega-García, M. E. García-Llamas, Z. Gutiérrez-Hervás, A. Sáez-Aguado, M. R. Villa-Carcedo, A. Álvaro-Prieto

**Affiliations:** 1 Psiquiatría y Salud Mental. Unidad de Caludad y Seguridad del Paciente, Hospital El Bierzo. GASBI. SACYL. CIBERSAM; 2 Área de Medicina Preventiva y Salud Pública. Depratamento de Ciencias Biomédicas, Universidad de León; 3 Psiquiatría y Salud Mental, Hospital El Bierzo. GASBI. SACYL, Ponferrada (León); 4 Junta de Castilla y León; 5 SERV. DE COORD. ASIS. SOCIOSANITARIA Y SALUD MENTAL, Gerencia Regional de Salud de Castilla y León. SACYL, Valladolid, Spain

## Abstract

**Introduction:**

Humanization in Mental Health is a concept that treat to conceal in the last decades the quality, efficiency and safety of care of complex diseases and conditions with individual values, needs and preferences and involves the patient and society in the decision-making priority.

**Objectives:**

to stablish and evaluate the priorities of different groups of interest in the development of a new humanization plan for mental health

**Methods:**

During 2022 a Humanization plan for the Spanish region of Castilla y Leon (2.400.000 habs) was developed with a Delphi model. Participants included 36 stakeholders including mental health services, administration, social services, associations, patients and families. They stablished 32 objectives distributed in 7 strategic lines: 1. “People First” (Rights, Autonomy and Information); 2. “From People to Services” (Participation of users in mental heal services); 3. “Person-Centered-Assistance” 4. “Processes sensible to change” (reduction of coercion); 5. “Human ambient” (Improvement of units, psychosocial interventions). 6. Innovation, training and climate (not evaluated here). 7. “People without marks” (battle against stigma).

Priorities in the lines were stablished by representatives from mental health and other healthcare professionals, social and educational stakeholders, scientific societies, patients and families. After agreeing to participate in the process, they had to answer an online survey. For each line, they have to score it from 0 to 10.

**Results:**

500 subjects participated (38.6% Healthcare workers, 14% Mental Health Care users, 9.8% Social Services, 8.8% Associations, 7.8% Drug Services 6% Management of Health System, 5.8% Education Services, 3.8 Justice). Humanization was the most appreciated plan within the mental health plan 2022-2026 in Castilla y Leon (8.81±1.43).

The Highest priority score was given to the Rights (8.68 + 1.54), Information (8.44 + 1.60) and Stigma (8.43 + 1.89) lines and the lowest were the evaluation of satisfaction (7.62 + 1.90) and Reduction of Coercion (7.29 + 2.12). Differences were found between groups. Scores in Rights and Autonomy (F:3.474; p<0.001) were highest in the Associations (9.32 +1.01) and lowest in the Justice group (7.68 + 1.67). In the information line the highest score (F:2.431; p=0,014) was in the Education Services (9.03 +0,94) compared to Scientific Societies (7,65 + 2,13). Highest score for Participation of Users (F:2,968; p=0,003) was in Social Services (8.76 +1.48) compared to Justice (7.47 +1.95). There were differences in the coercion reduction line (F:2.165; p=0,029) but no pairwise differences were found

**Image:**

**Figure:**
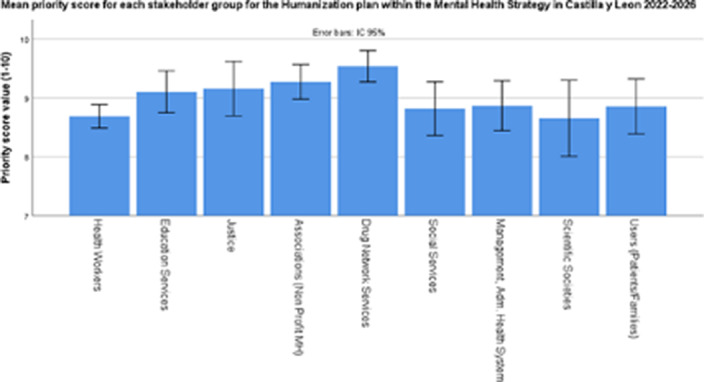

**Image 2:**

**Figure:**
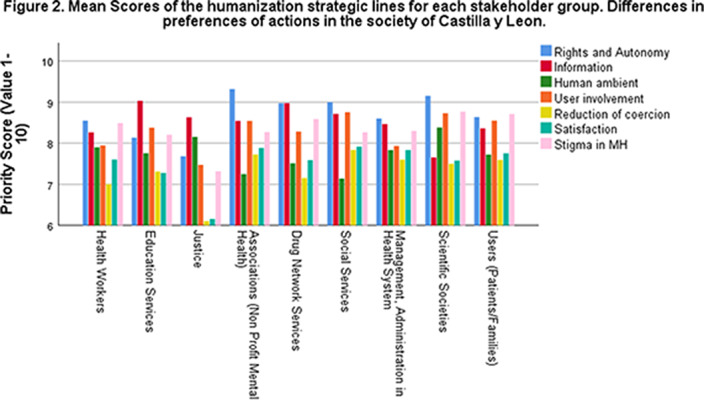

**Conclusions:**

Humanization approaches are well appreciated by different stakeholders. Priorities in our region start with rights, information and integration and mental health users in the health system and society

**Disclosure of Interest:**

None Declared

